# Hemoglobin Oxidation Reactions in Stored Blood

**DOI:** 10.3390/antiox11040747

**Published:** 2022-04-08

**Authors:** Abdu I. Alayash

**Affiliations:** Laboratory of Biochemistry and Vascular Biology, Division of Blood Components and Devices (DBCD), Center for Biologics Evaluation and Research (CBER), Food and Drug Administration (FDA), Silver Spring, MD 20993, USA; abdu.alayash@fda.hhs.gov; Tel.: +1-240-402-9350

**Keywords:** hemoglobin oxidation, blood storage, blood pathogen inactivation

## Abstract

Hemoglobin (Hb) inside and outside the red blood cells (RBCs) undergoes constant transformation to an oxidized form in a process known as autoxidation. The ferrous heme iron (Fe^2+^) of the prosthetic group is spontaneously transformed into an oxidized ferric (Fe^3+^) form, but under oxidative stress conditions a higher oxidation ferryl heme (Fe^4+^) is also formed. Although Fe^3+^ is a non-functional form of Hb, the Fe^4+^ is also extremely reactive towards other biological molecules due to its high redox potential. The RBC contains an effective reductive machinery that maintains Hb in the functional form with little oxidation during its life span. The redox transformation of Hb occurs to a lesser extent in young RBCs; it may, however, have detrimental effects on the integrity of these cells during ex vivo storage or when RBCs are subjected to pathogen reduction processes. In this review, Hb oxidation reactions (“oxidative lesion”) will be described, including details of how these reactions might impact the clinical use of stored or processed blood for therapeutic purposes.

## 1. Hemoglobin Oxidative Reactions and Toxicity

In the early 1980s, Hb was described as a biologic Fenton reagent that can facilitate the production of hydroxyl radicals ^•^OH from reactive oxygen species (ROS), and that these hydroxyl radicals are generated from the reaction of Hb’s iron with hydrogen peroxide (H_2_O_2_) [1]. In the last three decades, we have witnessed increased commercial efforts in transforming cell-free Hb to an oxygen-carrying (HBOC) therapeutic to be used in lieu of RBCs in transfusion medicine. Because of increasing opportunities for researchers to work on free Hb-based oxygen therapeutics, a more complete picture has emerged of Hb’s redox activity, resulting in Hb gaining a new “honorary enzyme” status as a pseudoperoxidase protein [2,3]. The pseudoperoxidative activity of Hb has been fully investigated in several acellular Hb solutions developed as blood substitutes with diverse origins and chemistries [3].

Central to this activity is the transformation of ferrous (HbFe^2^) to highly reactive intermediates. In this reaction, the oxidant H_2_O_2_ drives a catalytic cycle that includes three distinct steps: (1) initial oxidation of HbFe^2^ (oxy) to ferryl (HbFe^4+^) (accompanied by a ferryl protein radical), (2) autoreduction of the ferryl intermediate to ferric (HbFe^3+^) (met), and (3) reaction of metHb with an additional H_2_O_2_ molecule to regenerate the ferryl intermediate. Ferryl Hb is a highly reactive species that targets other biological molecules due to its high redox potential (E1/2° ~1.0 V). It can be detected by conventional spectrometric methods while the protein radical associated with the ferryl can only be measured by EPR [4].

Ferryl Hb was detected in several ex vivo and in vivo model systems: in atherosclerotic lesions of carotid arteries [5], in blood from mice and sickle cell disease (SCD) patients [6,7] and in blood from SCD patients infected with malaria [8]. Unique cellular, and in some instances subcellular, injuries have been attributed to the ferryl Hb’s redox reactivities. Collectively, these studies established, for the first time, a central role for cell-free Hb in cellular injury, and that these effects are mediated through the redox transition of Hb to higher oxidation states, leading ultimately to heme loss.

SCD is a unique model system that enabled the close investigation of Hb’s pseudoperoxidase activities [9]. Sickle cell Hb (HbS) showed profound changes when exposed to oxidants, where ferryl Hb persisted longer in solutions than ferryl HbA, and was therefore more damaging to tissues and organelles [10]. It was suggested that Hb-dependent oxidation reactions in stored blood may follow similar patterns to those observed in SCD RBCs. Although SCD RBCs have unique structural and rheological properties, they also share some common features with inactivated and/or stored RBCs such as structural membrane changes, reduced deformability, intracellular oxidation reactions and microparticle formation [11].

## 2. Current Blood Banking Practices and Blood Safety

The US blood supply is generally safe, although concerns remain about the existence of emerging pathogens that may compromise the safety of donated blood. RBCs are the most frequently transfused blood product, with approximately 11 million red cells transfused annually as part of the standard treatment to increase oxygen-carrying capacity and RBC mass [12].

RBCs are derived from either whole-blood donations or collected by apheresis technology. The volume of an average unit of packed RBCs ranges from 200 to 350 mL while hematocrit ranges from 55% to 80%. The shelf life of RBCs varies depending on the anticoagulant preservative used to store the product. The use of additive solutions allows for a longer shelf life. Generally, in the United States, RBC units in additive solutions can be stored for up to 42 days. One unit of RBCs is expected to raise the [Hb] of an adult patient by 1 g/dL or hematocrit by approximately 3% [12].

Some of the challenges with RBC storage and processing include determining the appropriate storage age of RBCs for transfusion and the use of pathogen inactivation technologies. The storage of RBCs results in physiologic changes measured by in vitro parameters, including impaired nitric oxide (NO) metabolism [13], the accumulation of lactic acid, decreased pH, ATP, and 2,3-DPG, the release of inflammatory mediators, and increased red cell membrane inflexibility [14]. Collectively, these metabolic changes are known as the “storage lesion”, which may contribute to impaired oxygen uptake and delivery [15].

Based on these in vitro studies it was believed that increased storage time would impair the quality of RBCs, but multiple randomized controlled trials evaluating the indications and outcomes of transfusion have been less definitive, providing contradicting evidence “for” or “against” transfusion strategies in adult patients [12]. The development of new technologies to refine the decision-making process for RBC transfusion, such as non-invasive methods of directly assessing tissue oxygenation as well as possible mitigation of the deleterious effects of storage, is currently an active area of research [16].

In addition to the need for blood collection facilities to maintain adequate supplies of blood, the military interest in a readily available product encouraged the development of alternatives to donated blood. Several oxygen-therapeutic products have been developed in recent years. Shelf-stable, portable, one-type-fits-all blood substitutes have long held theoretical promise as replacements for standard blood transfusions in extreme, life-threatening situations, such as trauma [3]. Safety concerns, however, have hampered the development and ultimate FDA approval of these products, in part due to the uncontrollable Hb redox side reactions. The focus of the following sections is on RBC storage, RBC pathogen inactivation technologies, and the potential effects on Hb oxidative stability using our past experiences with HBOCs.

## 3. Hemoglobin Oxidative Pathways in Stored Blood

Roughly 1–3% of Hb is normally transformed into an oxidized non-functional (ferric/met) form within RBCs during their normal lifespan. MetHb is rapidly converted back to ferrous/oxyHb by metHb reductase in the presence of NADH. During the storage of RBCs, the activity of metHb reductase is diminished, resulting in increased levels of metHb that is not converted back to oxyHb. The increase in Hb oxidation during storage of RBCs may also be due to a decrease in their antioxidant capacity, resulting in the oxidation and deterioration of membrane lipids and proteins, which can ultimately lead to irreversible damage to the membrane. During storage, the spontaneous lysis of a small fraction of red cells takes place and vesicles containing both lipids and Hb from intact red cells are shed into the supernatant plasma [17]. Although there is very limited information in the literature that specifically deals with Hb oxidation, or oxidative injury during hypothermic storage of RBCs, a possible link between Hb oxidation and the biopreservation of blood was recently noted [18,19].

The effects of RBC exchange transfusion doses (1, 3, and 9 units), storage period (14 days), and mortality were recently evaluated in a guinea pig model with a vascular disease phenotype. The guinea pig was chosen due to its similarity to humans in that it lacks ascorbate synthetic pathways, an important antioxidant mechanism known to control Hb oxidation reactions [20]. This study showed that increases in plasma non-transferrin-bound iron (NTBI) and Hb resulting from stored-blood transfusions contributed to vascular disease-associated mortality through iron-enhanced Hb oxidation and subsequent tissue injury [21].

Time-dependent oxidative challenges, including the formation of ROS, attachment of denatured Hb to membrane phospholipids and the release of Hb-containing membrane microvesicles throughout storage have been recently documented [18]. Other complications include an increased rate of Hb oxidation during the ex vivo storage of RBCs, compromised antioxidant activity, high concentrations of glucose in the storage media and the presence of molecular oxygen [18]. RBCs preserved in citrate–phosphate–dextrose–adenine storage solution units for up to 6 weeks exhibited oxidative injury characterized by the attachment of denatured Hb, presumably hemichromes, to membrane phospholipids and cytoskeleton proteins, such as spectrin [19]. Traces of denatured Hb were also present in microvesicles [22].

Better appreciation of RBC oxidative metabolism and the mechanism of storage damage (“oxidative lesion”) led to the development of better additive solutions and storage procedures, which extended the hypothermic storage limit to 6 weeks [23,24]. Attempts to minimize oxidative damages have recently been explored. It was reported that human plasma and tirilazad mesylate protected stored human RBCs from the oxidative damage of gamma irradiation [22]. Tirilazad mesylate, a nonglucocorticoid, 21-aminosteroid, inhibits lipid peroxidation by a combination of radical scavenging and membrane-stabilizing properties. It was noted that normal human plasma was effective at protecting against the oxidative damage of irradiated RBCs. Furthermore, the synthetic antioxidant trilazad mesylate was effective at protecting red cells against oxidative damage as it scavenges ROS. In some studies, it was even suggested that antioxidants be either given to the donor before donation or added to the blood bag [22].

The role of nitric oxide (NO) redox transformation by Hb spilled over into blood transfusion practices as a potential intervention strategy to reverse the storage lesion. Biochemical processes such as the S-nitrosylation (SNO) of βCys93 or nitrite reductase enzymatic activity of Hb resulted in Hb/NO complexes/intermediates that can release NO and reverse biochemical changes associated with RBC aging [25,26]. NO binds to Hb at rates almost 600 times higher than its natural partner, oxygen. The most instantaneous byproduct of this reaction is metHb-NO complexes that can be redox cycled by Hb with little or no damage to the protein [27].

The release of NO from Hb within RBCs is kinetically and physiologically unlikely to occur [28,29].

## 4. Pathogen Inactivation of Blood

The development of methods of pathogen reduction will remain an active area of research in transfusion medicine [30]. As mentioned earlier, blood supplies in the US are generally safe, but emerging pathogens still represent a challenge. The risk of viral infections from transfusion was calculated to be about 1:1,900,000 for HIV, 1:137,000 for HBV, and 1:1,000,000 for HCV. Thus, the risk from viral infection is not zero and bacterial contamination also remains a major concern [30]. The terms pathogen inactivation (complete prevention of infection by a pathogen) and pathogen reduction (decreasing the amount of infectivity of a pathogen) have been used interchangeably [31].

The two major approaches to pathogen inactivation involve methods that inactivate lipids, thus targeting many pathogenic lipid-enveloped viruses and methods that damage nucleic acids. Most current methods are based on DNA intercalators: the product psoralen, manufactured by Intercept, and riboflavin, manufactured by Mirasol. These technologies both require UV irradiation to activate the intercalators and to inactivate the pathogens’ nucleic acids. The use of UV irradiation, however, tends to reduce the efficacy of the transfusable components of whole blood, as well as packed RBCs [32].

Current commercial pathogen reduction and/or inactivation processes are largely based on exposing major blood components (RBCs, platelets, and plasma) to UV light and photosensitizer molecules. For platelets and plasma, pathogen reduction technologies are routinely carried out in many blood establishments. For RBCs, and particularly with more recent searches for ideal strategies to inactivate whole blood (i.e., effective in microbial killing while gentle on the transfusion component), the identification and development of a new generation of pathogen reduction processes will be an area of active investigation [33].

The intercalator, S-303 (amustaline), was specifically developed by Cerus Corporation for the pathogen inactivation of RBCs and does not require UV light activation [31]. S-303 is a modular compound that prevents nucleic acid replication by targeting and cross-linking nucleic acids. Once added to the RBC unit, this amphipathic compound rapidly passes through cell and viral envelope membranes and intercalates into the helical regions of nucleic acids. S-300, the non-reactive byproduct of this reaction, is subsequently removed by incubation and centrifugation [31].

Glutathione (GSH), a naturally occurring antioxidant, must be added to RBC solutions to prevent non-specific reactions between S-303 and other nucleophiles present in the RBC unit. However, these thiol reagents are known to react with cysteine β93 of Hb, leading to changes in the oxygen affinity as well as oxidative stability of the protein [4,34]. A second-generation S-303 pathogen inactivation process to improve on the first-generation S-303 procedure only marginally affected RBC quality and function by increasing the quencher concentration of GSH.

**Figure 1:**
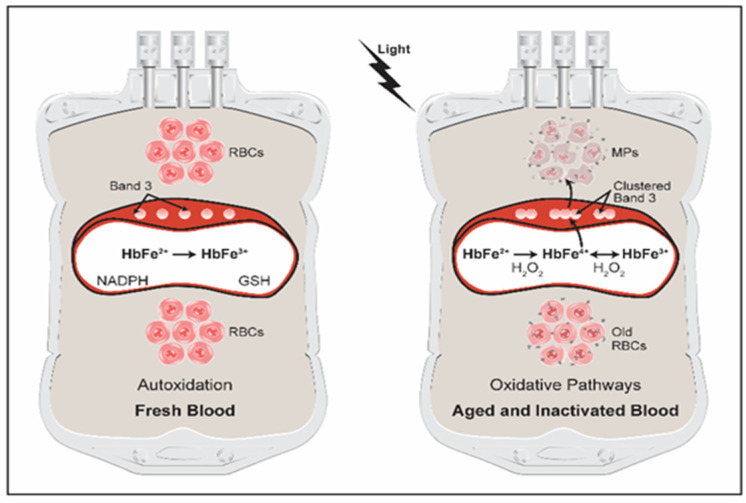
RBC oxidative injury during storage and pathogen inactivation conditions. Freshly stored RBCs in a standard blood bag undergo very little oxidation apart from normal spontaneous (autoxidation) reactions of Hb, resulting in little metHb accumulation (left). Reductive and antioxidant enzymes/proteins such as NADPH reductase and GSH maintain metHb to a minimum. Under prolonged storage conditions or when RBCs are exposed to UV light, Hb oxidative side reactions are increased, mainly Hb’s own pseudoperoxidative pathways (right). These pathways result in the production of ferryl Hb (HbFe^4+^) which attacks other biological targets including band 3, resulting in band 3 clustering. Ferryl Hb crosslinks the major RBC membranes band 3 into clusters and the ultimate release of Hb-laden microparticles (MPs), based on [18,37] with modifications.

Circulating RBCs are subject to a continuous onslaught of endogenous and exogenous oxidative stress, and expectedly more stress is incurred during storage and during the harsh treatment by chemicals and/or exposure to UV lights [35]. Unintended consequences impacting the integrity of RBCs have hindered the development of safe and effective methods. The UV light radiation of blood contained in plastic bags may lead to the deterioration of RBCs [36]. The participation of ROS may drive some of the observed biochemical changes that included cytosolic proteins (including Hb) and lipid proteins [33]. The molecular mechanisms leading to the deterioration of RBCs are not well defined. Current pathogen inactivation methods play an important part in promoting an oxidative milieu which may promote Hb oxidative side reactions that impact the biopreservation of blood [33]. The role of Hb in these reactions is therefore critical as Hb itself can be a source of ROS. Hb can also avidly react with ROS, transforming itself into a highly reactive and disruptive agent [3,4] (see Figure 1). Current research activity is focusing on the understanding of the origins of these reactions along with investigating methods to minimize them.

## 5. Hemoglobin Oxidation Reactions Drive Changes within RBCs Resulting in Microparticles Formation

It was recently noted that increases in storage time compromise pathways responsible for the regeneration of the essential reducing equivalent NADPH and the dysregulation of metabolon assembly at the cytoplasmic domain of band 3 membrane proteins [38]. Band 3 is a major RBC membrane-spanning protein involved in ion transport and protein–protein interactions. Clustering of band 3 was shown by our group to be driven by cytosolic Hb oxidation, which ultimately led to microparticle (MPs) formation [6,7] (Figure 1).

MPs are small phospholipid vesicles less than 1 μm in diameter that are released into the bloodstream from a variety of cells, including RBCs. Initially, they were considered as cell fragments or “dust” without any biological function. However, MPs are now recognized as being involved in a number of clinical and physiological situations, including RBC storage lesion associated with transfused blood stored for more than 21 (or 30) days [39].

There are two main mechanisms believed to be responsible for the formation of MPs during RBC storage, including a process known as eryptosis (similar to the apoptosis of nucleated cells, RBCs may enter suicidal death, eryptosis) that leads to microvesiculation, or band 3 clustering [6] (Figure 1). Both events are accompanied by phosphatidylserine externalization on the RBC membrane and the degradation of cytoskeleton proteins followed by modifications in the phosphorylation status of band 3, resulting in MP formation and release [6].

Because of the high Hb content within MPs, this may result in NO scavenging and pro-coagulant activity enhancement and could therefore significantly affect NO bioavailability. As mentioned earlier, aged RBCs share a number of features with sickle RBCs that include cytosolic, mainly Hb, and membrane changes, leading to RBC sickling and vesiculation [40]. The levels of MPs have also been shown to be elevated both in a steady state and during vaso-occlusive crisis [41,42].

We have recently carried out studies in blood from a sickle cell mouse model and from SCD patients, specifically investigating the role of Hb oxidation in MP formation and toxicity. These oxidative changes ultimately result in MPs releasing their highly oxidizing cargo (Hb) outside these vesicles. We used photometric and proteomic methods that confirmed the presence of high levels of Hb oxidation intermediates (ferric/ferryl), resulting in β-globin post-translational modifications, including the irreversible oxidation of βCys93 and the ubiquitination of βLys96 and βLys145. The high levels of ferryl Hb content within MPs were correlated with complex formation with band 3 and RBC membrane proteins (Figure 1). The incubation of Townes-SS MPs for instances with human endothelial cells caused a greater loss of monolayer integrity, apoptotic activation, heme oxygenase-1 induction, and concomitant bioenergetic imbalance compared with control Townes-AA MPs [6,7].

## 6. Conclusions

In conclusion, based on the extensive knowledge accumulated over the last few decades related to the damaging oxidative pathways of free Hb and intraerythrocytic Hb (in health and in disease states), we can now fully explore these reactions in situations where RBCs are stored for a long duration, inactivated or even when derived from stem cells. The redox-dependent cytosolic and membrane changes inside aging RBCs, and when RBCs are subjected to harsh treatment with UV, must be fully understood in order to design specific countermeasures.
